# Multi-region investigation of ‘man’ as default in attitudes

**DOI:** 10.1371/journal.pone.0323938

**Published:** 2025-06-25

**Authors:** Curtis Edward Phills, Jeremy K. Miller, Erin M. Buchanan, Amanda Williams, Chanel Meyers, Elizabeth R. Brown, Janis Zickfeld, Selina Volsa, Stefan Stieger, Elisabeth Oberzaucher, Vinka Mlakic, Martin Vasilev, İlker Dalgar, Sami Çoksan, Sinem Söylemez, Çağlar Solak, Asil Ali Özdoğru, Belemir Çoktok, Chun-Chia Kung, Panita Suavansri, Harry Manley, Sara Álvarez-Solas, Danilo Zambrano Ricaurte, Ivan Ropovik, Gabriel Baník, Peter Babinčák, Matúš Adamkovič, Pavol Kačmár, Monika Hricová, Jozef Bavoľár, Lisa Li, Fei Gao, Zhong Chen, Vanja Ković, Vasilije Gvozdenović, Patrícia Arriaga, Katarzyna Filip, Krystian Barzykowski, Sylwia Adamus, Gerit Pfuhl, Sarah E. Martiny, Kristoffer Klevjer, Frederike S. Woelfert, Christian K. Tamnes, Jonas R. Kunst, Max Korbmacher, Margaret Messiah Singh, Sraddha Pradhan, Noorshama Parveen, Arti Parganiha, Babita Pande, Pratibha Kujur, Priyanka Chandel, Niv Reggev, Aviv Mokady, Marietta Papadatou-Pastou, Roxane Schnepper, Jan Philipp Röer, Tilli Ripp, Ekaterina Pronizius, Claus Lamm, Martin Voracek, Jerome Olsen, Janina Enachescu, Carlota Batres, Daniel Storage, Carmel A. Levitan, Manyu Li, Leigh Ann Vaughn, William J. Chopik, Kathleen Schmidt, Peter R. Mallik, Savannah Lewis, Brynna Leach, Brianna Jurosic, David Moreau, Izuchukwu Lawrence Gabriel Ndukaihe, Nwadiogo Chisom Arinze, Steve M. J. Janssen, Alicia Foo, Chrystalle B. Y. Tan, Glenn P. Williams, Danny Riis, Bethany M. Lane, Dermot Lynott, Thomas Rhys Evans, Miroslav Sirota, Dawn L. Holford, Kaitlyn M. Werner, Kelly Wang, Marina Milyavskaya, Ian D. Stephen, Robert M. Ross, Andrew Roberts, Omid Ghasemi, Niklas K. Steffens, Kim Peters, Barnaby Dixson, Marco Antonio Correa Varella, Jaroslava V. Valentova, Anthonieta Looman Mafra, Rafael Ming Chi Santos Hsu, Yago Luksevicius de Moraes, Luana Oliveira da Silva, Caio Santos Alves da Silva, Mai Helmy, Mariah Balderrama, Ali H. Al-Hoorie, Tyler McGee, Zahir Vally, Attila Szuts, Patrick Forscher, Pablo Bernabeu, Balazs Aczel, Anna Szabelska, Sau-Chin Chen, Christopher R. Chartier, Zoltan Kekecs

**Affiliations:** 1 Department of Psychology, University of North Florida, Jacksonville, Florida, United States of America; 2 Department of Psychology, University of Oregon, Eugene, Oregon, United States of America; 3 Department of Psychology, Willamette University, Salem, Oregon, United States of America; 4 Analytics, Harrisburg University of Science and Technology, Harrisburg, Pennsylvania, United States of America; 5 Waterloo Regional Police Service, Cambridge, Ontario, Canada; 6 Department of Management Aarhus University, Aarhus, Denmark; 7 Department of Psychology and Psychodynamics, Karl Landsteiner University of Health Sciences, Krems an der Donau, Austria; 8 Department of Evolutionary Anthropology, University of Vienna, Wien, Austria; 9 Bournemouth University, Talbot Campus, Poole, United Kingdom; 10 Department of Psychology, Ankara Medipol University, Ankara, Turkey; 11 Department of Psychology, Erzurum Technical University, Erzurum, Turkey; 12 Network for Economic and Social Trends, Western University, London, Ontario, Canada; 13 Department of Psychology, Manisa Celal Bayar University, Manisa, Turkey; 14 Department of Psychology, Marmara University, İstanbul, Turkey; 15 Department of Psychology, Üsküdar University, İstanbul, Turkey; 16 Department of Psychology, National Cheng Kung University, Tainan, Taiwan; 17 Faculty of Psychology, Chulalongkorn University, Bangkok, Thailand; 18 Faculty of Behavioral Sciences, Education, and Languages, HELP University Subang 2, Shah Alam, Malaysia; 19 Ecosystem Engineer, Universidad Regional Amazónica Ikiam, Tena, Ecuador; 20 Faculty of Psychology, Fundación Universitaria Konrad Lorenz, Bogotá, Colombia; 21 Institute for Research and Development of Education, Faculty of Education, Charles University, Prague, Czechia; 22 Faculty of Education, University of Presov, Presov, Slovakia; 23 Institute of Psychology, University of Presov, Presov, Slovakia; 24 Pavol Jozef Safarik University, Kosice, Slovakia; 25 Institute of Social Sciences, CSPS, Slovak Academy of Sciences, Bratislava, Slovakia; 26 University of Jyväskylä, Jyväskylä, Finland; 27 Department of Psychology, Faculty of Arts, Pavol Jozef Šafarik University in Košice, Košice, Slovakia; 28 Faculty of Arts and Humanities, University of Macau, Macau, China; 29 Laboratory for Neurocognition and Applied Cognition, Faculty of Philosophy, University of Belgrade, Belgrade, Serbia; 30 Iscte-University Institute of Lisbon, CIS-IUL, Lisbon, Portugal; 31 Institute of Psychology, Jagiellonian University, Krakow, Poland; 32 Department of Psychology, UiT-The Arctic University of Norway, Tromsø, Norway; 33 Department of Psychology, Norwegian University of Science and Technology, Trondheim, Norway; 34 Department of Psychology, University of Oslo, Oslo, Norway; 35 Department of Health and Functioning, Western Norway University of Applied Sciences, Bergen, Norway; 36 School of Studies in Life Science, Pt. Ravishankar Shukla University, Raipur, India; 37 Department of Psychology and School of Brain Sciences and Cognition, Ben Gurion University of the Negev, Beer Sheba, Israel; 38 School of Education, National and Kapodistrian University of Athens, Athens, Greece; 39 Department of Psychology and Psychotherapy, Witten/Herdecke University, Witten, Germany; 40 Department of Cognition, Emotion, and Methods in Psychology, Faculty of Psychology, University of Vienna, Vienna, Austria; 41 Faculty of Psychology, University of Vienna, Wien, Austria; 42 Department of Psychology, Franklin and Marshall College, Lancaster, Pennsylvania, United States of America; 43 Department of Psychology, University of Denver, Denver, Colorado, United States of America; 44 Department of Cognitive Science, Occidental College, Los Angeles, California United States of America; 45 Department of Psychology, University of Louisiana at Lafayette, Lafayette, Louisiana, United States of America; 46 Department of Psychology, Ithaca College, Ithaca, New York, United States of America; 47 Department of Psychology, Michigan State University, East Lansing, Michigan, United States of America; 48 Department of Psychology, Ashland University, Ashland, Ohio, United States of America; 49 Hubbard Decision Research, Glen Ellyn, Illinois, United States of America; 50 Department of Psychology, University of Alabama, Tuscaloosa, Alabama, United States of America; 51 School of Psychology, University of Auckland, Auckland, New Zealand; 52 Department of Psychology, Alex Ekwueme Federal University, Ndufu-Alike, Nigeria; 53 School of Psychology, University of Nottingham Malaysia, Selangor, Malaysia; 54 School of Psychology and Vision Sciences, University of Leicester, Leicester, United Kingdom; 55 Faculty of Health Sciences and Wellbeing, School of Psychology, University of Sunderland, Sunderland, United Kingdom; 56 Division of Psychology, School of Social and Health Sciences, Abertay University, Dundee, United Kingdom; 57 Department of Psychology, Maynooth University, Maynooth, Ireland; 58 School of Social, Psychological and Behavioural Sciences, Coventry University, Coventry, United Kingdom; 59 Department of Psychology, University of Essex, Colchester, United Kingdom; 60 Department of Psychology, Carleton University, Ottawa, Canada; 61 Department of Psychology, Macquarie University, Sydney, Australia; 62 Department of Psychology, Nottingham Trent University, Nottingham, United Kingdom; 63 Department of Philosophy, Macquarie University, Sydney, Australia; 64 School of Psychology, University of New South Wales, Kensington, Australia; 65 School of Psychology, University of Queensland, Brisbane, Australia; 66 School of Health Psychology, University of the Sunshine Coast Queensland, Brisbane, Australia; 67 Department of Experimental Psychology, Institute of Psychology, University of Sao Paulo, São Paulo, Brazil; 68 Department of Psychiatry, Faculty of Medicine, University of São Paulo, São Paulo, Brazil; 69 Psychology Department, College of Education, Sultan Qaboos University, Muscat, Oman; 70 Royal Commission for Jubail and Yanbu, Jubail, Saudi Arabia; 71 Department of Clinical Psychology, United Arab Emirates University, Al Ain, United Arab Emerates; 72 Institute of Psychology, ELTE, Eotvos Lorand University, Budapest, Hungary; 73 LIP/PC2S, Université Grenoble Alpes, Grenoble, France; 74 Busara Center for Behavioral Economics, Nairobi, Kenya; 75 Department of Psychology, Lancaster University, Lancaster, United Kingdom; 76 Department of Language and Culture, UiT The Arctic University of Norway, Tromsø, Norway; 77 Institute of Cognition and Culture, Queen’s University Belfast, Belfast, United Kingdom; 78 Department of Human Development and Psychology, Tzu-Chi University, Hualien, Taiwan; Cardiff University, UNITED KINGDOM OF GREAT BRITAIN AND NORTHERN IRELAND

## Abstract

Previous research has studied the extent to which men are the default members of social groups in terms of memory, categorization, and stereotyping, but not attitudes which is critical because of attitudes’ relationship to behavior. Results from our survey (*N* > 5000) collected via a globally distributed laboratory network in over 40 regions demonstrated that attitudes toward Black people and politicians had a stronger relationship with attitudes toward the men rather than the women of the group. However, attitudes toward White people had a stronger relationship with attitudes toward White women than White men, whereas attitudes toward East Asian people, police officers, and criminals did not have a stronger relationship with attitudes toward either the men or women of each respective group. Regional agreement with traditional gender roles was explored as a potential moderator. These findings have implications for understanding the unique forms of prejudice women face around the world.

## Introduction

When asked to imagine a person, people tend to think of a man [1]. Similarly, many social categories like ‘politician’ are gendered in that group stereotypes are ascribed more strongly to the men, rather than the women of the group [2]. Many racial groups (e.g., Black people) are also gendered in the sense that the group’s men are seen as the default [3]. These effects are consistent with androcentrism, the belief that men are the default and women the exception or ‘other’ [4–6]. Previous research has studied the extent to which men are the default members of social groups in memory [7], categorization [8], and stereotyping [9]. The present research builds upon this literature by studying ‘man’ as default in attitudes which is important because of attitudes’ relationship to behavior [10]. We recruited a large, racially diverse sample of participants through a global distributed laboratory network, the Psychological Science Accelerator (PSA) [11], to examine whether men were default in attitudes toward traditionally male- (politicians, police, criminals) and race-based (Black people, White people, East Asian people) groups.

### ‘Man’ as default in attitudes

In English, the word ‘people’ is ostensibly gender-neutral in that it could refer to persons of any gender. However, in practice, people write about ‘people’ as though they were men [12]. The same is true for some racial groups (e.g., White) in that people write about them as though the group primarily consists of men [13].

Assuming men are the ‘default’ has negative consequences because it prioritizes their experiences and ideals over those of women [4]. For example, women are less motivated to apply for jobs that use ‘he’ pronouns to describe the ideal candidate [14]. The assumption that men are the primary sufferers of heart disease, when it is actually more common in women [15], leads to women not receiving treatment until they are older and sicker [16]. A policewoman was stabbed to death while operating a hydraulic ram because her protective armor was designed for the ‘average man’ and, therefore, did not fit properly [17].

Important to the present research, assuming men are the ‘default’ members of higher status groups like politicians and police has negative consequences for attitudes toward women who are members of those groups. Specifically, role congruity theory [18,19] argues that prejudice is partially rooted in the perceived misalignment between the characteristics typically ascribed to women (communal traits) and the qualities desired of an occupant of a traditionally male role (agentic traits) [20,21]. For example, women in leadership positions face backlash partly because they threaten the gender status quo [22]. Thus, it is not surprising that in the United States only 29% of state legislative seats are held by women [23] and around the world among UN member states only 26% of parliamentarians are women [24]. Similarly, in the United States only 14% of police officers are women [25] though this percentage ranges from 9% in Portugal to over 60% in Sweden [26].

Notably, men can be ‘default’ in lower status groups as well. For example, 93% of incarcerated people in U.S. federal prisons [27] and around the world [28] are men. However, the number of incarcerated women has increased by sixty percent this century. For example, in Brazil four times as many women were incarcerated in 2022 compared to 2000 [28]. Thus, though there could be regional variation, attitudes toward traditionally male groups, regardless of the status of those groups, should be more similar to attitudes towards the group’s men rather than women because attitudes toward women in those roles are, partially, rooted in misogynistic beliefs about the traits typically ascribed to women (i.e., communal traits) and the traits desired in people performing traditionally male roles (i.e., agentic traits). This pattern could be moderated by the extent to which a region enforces traditional gender roles because strict enforcement of traditional gender roles strengthens the incongruity of a woman in a traditionally male role.

### ‘Man’ as default in attitudes toward racial groups

The intersection of race and gender often means that the men of a racial group are considered the default [29]. For example, people tend to imagine a man when asked to think about a Black person or a White person [3]. Similarly, Black men are categorized as Black faster than Black women [8]. In regard to racial stereotypes, one study found that 11 of the 15 most frequent stereotypes participants listed about Black men overlapped with the most frequent stereotypes listed about Black people, but only five of the 15 most frequent stereotypes listed about Black women overlapped with the most frequent stereotypes listed about Black people [9]. Moreover, in certain contexts negative attitudes (or prejudice) is often directed at the men rather than the women of outgroups [30].

However, ‘man’ may not be the default for some racial groups. For example, in the United States, people mis-remember conversational contributions by an Asian man more than an Asian woman, and they are less likely to think of a man when imagining an Asian person compared to when imagining a White person [3]. This may be because mental representations of race are gendered [8] or reflect the historical goals of a region’s dominant majority group [31]. Thus, for many people in the United States, East Asian women come to mind quicker than East Asian men.

Not only is it important to consider the gender of the target but also the gender of the participant [30], when studying the default gender in attitudes toward racial groups. For example, Black women are unlikely to exclude themselves from their representation of Black people. Thus, though we predicted that attitudes toward racial groups would be more similar to attitudes toward the men rather than the women of each racial group, we also predicted exceptions based on region (i.e., attitudes toward East Asians in the United States) and group membership (i.e., the women of each racial group).

### Overview

The present research investigated ‘man’ as default in attitudes by testing whether attitudes toward a social group were more strongly related to attitudes toward the men or women of the group. We predicted that for both traditionally male groups (politicians, police officers, criminals) as well as racial groups (Black people, White people, East Asian people), attitudes toward the group would be more strongly related to attitudes toward the men of the group than attitudes toward the women of the group. Predictions related to racial groups were tested on sub-samples that did not include members of that group. However, because research in the U.S. suggests that White participants are not more likely to imagine a man when thinking about East Asians [3], we predicted that among U.S. participants who were not East Asian, attitudes toward the group would not be more strongly related to attitudes toward the group’s men.

Notably, we predicted Black women, White women, and East Asian women would not view ‘man’ as the default member of their respective racial groups in attitudes.

## Method

Our predictions and research methods were pre-registered (https://osf.io/w4q6t/) and we note that we deviated from the pre-registration by not excluding participants who were police officers or politicians from the sample when testing predictions about those groups. This was done because the final version of the survey did not ask participants to identify whether they were police officers or politicians. In addition to our pre-registered hypotheses, we also explored the possibility that ‘man’ as default in attitudes would be moderated by the extent to which a region enforces traditional gender roles. Specifically, we predicted that the more a region endorsed traditional gender roles, the more strongly participants in that region would associate attitudes toward traditionally male groups more with attitudes toward the men rather than the women of that group.

### Participants

Participants (*N* = 5803; 5551 in lab, 252 online; age range 18–67, median age = 20) were recruited through the Psychological Science Accelerator (PSA) from over 40 regions around the world. Full details about running the study, including data preparation and pre-registration, are stored on the open science framework (https://osf.io/w4q6t/). Of those that started the experiment, 5,177 participants provided partial or complete data to be analyzed (72% women, 25% men, < 1% non-binary trans, 3% other or unknown; 56% White, 5% Black, 14% East Asian, 24% other or unknown race). See Table 1 for a breakdown of participant characteristics by region, race, and gender. Each laboratory either obtained approval from their local/institutional Ethics Committee or IRB, indicated that their institution did not require approval to conduct this type of study according to the rules of their area, or explicitly indicated that the current study was covered by a pre-existing approval. Related documents are stored on the open science framework (https://osf.io/bdycf/). Data collection began on April 15, 2019 and ended June 15, 2021.

**Table 1:** Sample size by region, gender, and race.

Region	N	Women	Men	Non Binary or Trans	Other or Unknown Gender	White	Black	East Asian	Other or Unknown Race
Australia	316	211	81	2	22	90	3	44	179
Austria	203	157	43	1	2	185	0	3	15
Brazil	74	48	23	0	3	49	3	0	22
Canada	105	57	43	0	5	39	19	14	33
China	53	29	21	0	3	0	0	51	2
Colombia	70	49	21	0	0	28	1	0	41
Ecuador	73	42	28	0	3	1	1	0	71
Germany	65	54	7	2	2	59	0	0	6
Greece	264	181	61	0	22	233	0	0	31
Hong Kong	20	16	3	0	1	0	0	19	1
Hungary	170	144	22	0	4	160	0	1	9
India	88	60	27	0	1	0	0	1	87
Israel	166	129	29	0	8	104	1	0	61
Macao	37	14	21	0	2	0	0	34	3
Malaysia	160	105	43	0	12	0	0	95	65
New Zealand	331	254	64	3	10	139	0	74	118
Nigeria	74	41	33	0	0	0	70	2	2
Norway	163	110	52	1	0	135	3	4	21
Poland	132	94	34	0	4	110	0	0	22
Portugal	65	35	29	0	1	59	0	0	6
Slovakia	316	283	29	0	4	307	0	0	9
Taiwan	219	128	88	0	3	1	1	192	25
Thailand	50	39	11	0	0	0	0	15	35
Turkey	286	236	48	0	2	33	2	2	249
United Kingdom	221	167	51	1	2	153	24	18	26
United States	1,422	1,012	374	12	24	923	127	160	212

Note. Regions with fewer than 10 participants are omitted. Many participants from Turkey identified as an ethnicity that could be considered ‘White’ but we categorized them as ‘Other’ because our predictions involving group membership are related to identity.

### Procedure

To make better use of the PSA’s globally distributed laboratory network, the current study was presented to participants in combination with an experiment related to the object orientation effect (https://osf.io/e428p/), and the stopping rules for data collection were related to that experiment. Specifically, the first part of the object orientation study was always presented to participants first and participants provided electronic consent to both studies during this phase by reading and agreeing to an informed consent document presented via a computer screen. The second part of the object orientation study and the current study were presented to participants in a random order such that the current study was either the second or third task participants completed. During the pandemic, 252 participants completed this study online as a standalone experiment.

In the current study, participants evaluated seven social groups (people, Black people, East Asian people, White people, police officers, politicians, and criminals) plus the women and men of each group in random order. Demographics (including age, race, gender), and debriefing were administered at the conclusion of the study.

### Group evaluation

Participants were instructed to ‘answer questions about social groups’ and to ‘respond openly and honestly.’ In accordance with research demonstrating that the best way to measure explicit racial attitudes is to directly ask about them [32], participants reported their attitudes by responding to four questions: how warm, positive, and favorable they felt towards each social group, as well as how much they liked each social group. The sliders could be moved to any value between 0 (*not at all*) and 100 (*completely*). Anchor points were displayed every 10 places (i.e., 10, 20, 30, etc.); however, participants were free to place the slider at any whole number between 0 and 100. The exact value participants chose was displayed on screen to the right of the slider. The social groups, as well as the questions related to each group, were shown to participants in a random order. Participants could skip any question they did not want to answer. A sample of the survey with the different translations can be found among the materials on OSF (https://osf.io/bdycf/).

As pre-registered, because items were highly correlated (*r*s > .81), a mean attitude score for each group was calculated by averaging responses on the four questions directed at the group. Mean scores for each group are presented in Table 2.

**Table 2:** Mean attitudes by social group and gender.

Category	Group Mean	Group SD	Women Mean	Women SD	Men Mean	Men SD
Black People	73.51	22.28	75.66	21.67	68.91	23.41
Criminals	16.42	19.87	20.84	22.82	15.00	19.39
East Asians	71.40	23.24	72.98	22.88	67.28	24.12
People	71.03	22.31	80.84	18.62	66.15	23.46
Police	52.72	29.58	53.66	29.40	61.17	28.23
Politicians	35.19	24.36	52.43	27.96	35.17	25.73
White People	73.07	21.97	69.29	22.98	63.80	25.38

Note. *N* > 5,000 for all groups. Scales ranged from 0 to 100 with higher scores indicating more positive attitudes.

### Creating relative attitude scores for each group

A relative attitude score for each target group was calculated by subtracting the mean attitude score for each group from the mean attitude score for people, men, or women, respectively. For example, relative attitudes toward Black people were calculated by subtracting attitudes toward Black people from attitudes toward people. Thus, relative attitude scores reflect how much more or less positive attitudes toward the target group are compared to attitudes toward the comparison group (i.e., people, men, or women) with higher scores representing higher prejudice against a group. See Table 3 for relative attitudes scores toward each group.

**Table 3:** Relative attitudes by social group.

Group	Attitudes toward Group people		Attitudes toward Group women		Attitudes toward Group men	
	*M*_*D*_	*SD*_*D*_	*M*_*D*_	*SD*_*D*_	*M*_*D*_	*SD*_*D*_
Black people	−2.48	23.93	5.20	17.31	−2.75	25.32
White people	1.75	21.40	7.76	18.54	2.36	17.22
East Asian people	−0.37	24.91	7.90	19.57	−1.15	26.23
Politicians	35.88	27.18	28.42	28.21	30.97	27.28
Criminals	54.63	28.87	60.00	28.26	51.15	29.43
Police officers	17.39	30.67	19.68	29.07	13.45	27.74

Note. Higher scores represent increased prejudice against a group. *N* > 5,000 for all groups.

### Regional endorsement of traditional gender roles

Data related to the extent a region endorsed traditional gender roles was obtained from the World Values Survey [33]. Participants in that survey answered questions about a variety of topics including four relevant to the present research: “Being a housewife is just as fulfilling as working for pay”; “On the whole, men make better political leaders than women do”; “A university education is more important for a boy than for a girl”; “On the whole, men make better business executives than women do” (1-agree to 4-disagree, reversed scored so that higher scores indicate stronger endorsement of traditional gender roles). The World Values Survey provided information about endorsement of traditional gender roles in twenty regions included in our dataset.

### Treating missing data

If a participant answered at least one of the four questions related to a group then we calculated the attitude score based on the available responses. Specifically, if only a single one of the four questions are answered, we used its score as the attitude score. However, if more than one question was answered, we calculated the average.

### Statistical analysis

A simulation-based power analysis demonstrated that 2,300 participants provided 90% statistical power in our design. Therefore, we conducted the planned confirmatory tests of our hypotheses when the relevant sub-sample for each hypothesis reached 2,300 participants. If we did not reach 2,300 participants for a particular sub-sample then we reported descriptive statistics and reported differences in correlation sizes (Pearson correlation coefficient) with 99.5% confidence interval, but we did not perform statistical tests nor draw any statistical inferences.

We tested for differences in the Group-Women and Group-Men correlations using the *cocor* package in *R* [34]. If the Group-Women correlation is.7 and the Group-Men correlation is.8, then the 99.5% confidence interval will be calculated around.1. In our analyses, we used the confidence intervals for statistical inference, always using a 99.5% interval, calculated using Zou’s [35] formula as implemented in the *cocor* package. If the confidence interval fell within *r* = −.1 and *r* = .1, we concluded that the difference in correlation size was not large enough to be of interest. Otherwise, if the confidence interval did not include 0, we concluded either that the prejudice against the group in general is more strongly correlated with prejudice against the women of that group than men of that group if the lower bound of the confidence interval was negative, or vice versa if the upper bound of the confidence interval was positive. If the confidence interval included both 0 and either −.1 or.1, we concluded that the test yielded an inconclusive result regarding the hypothesis, not supporting either the presence, or the absence of an effect.

## Results

**Fig 1:**
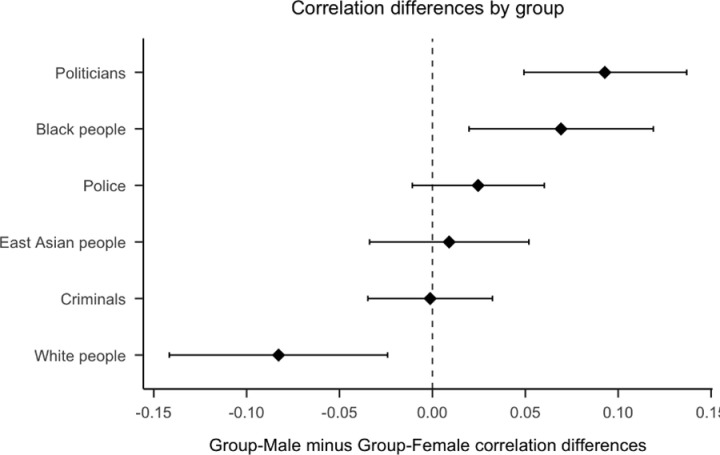
Plot of correlation differences by group. Positive scores indicate attitudes toward the group had a stronger correlation with attitudes toward the men rather than the women of the group. 99.5% confidence intervals calculated using the method outlined by Zou [35].

As per our pre-registered analysis plan (https://osf.io/3gux4/), we conducted confirmatory analyses on the first 2,300 participants to complete the study in each sub-sample (i.e., full sample, each racial group, the women of each racial group). Exploratory analyses were conducted on data from all participants who completed the study. In the descriptions below, we indicate whether we are conducting confirmatory hypothesis testing or exploratory analyses. The final analysis code is stored on the open science framework (https://osf.io/cj4tx). Fig 1 summarizes the findings presented in the next two sections.

To assess robustness, we conducted sensitivity analyses by running the analyses with each of the attitude questions (warm, positive, favorable, and likeability) separately, to see if any of these specific ratings are the main driver behind the results. However, none of the findings differed substantially from the ones run with the main aggregated attitude scoring presented below. These results do not change the conclusions of the study (see the numerical results in the supplemental analysis on OSF (https://osf.io/w4q6t)).

### ‘Man’ as default in attitudes toward traditionally male groups

**Fig 2:**
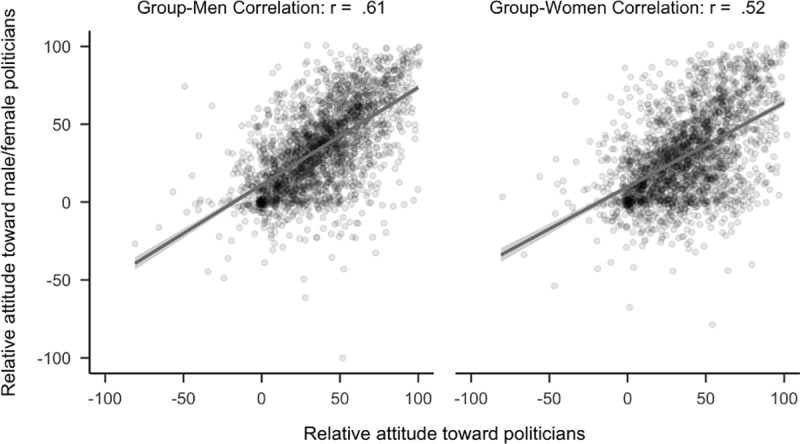
Plot of correlational differences for politicians. Side by side comparison of how relative attitudes toward politicians correlates with attitudes toward politicians who are women versus men. The sample is the first 2,300 participants to complete the study.

As predicted, a confirmatory hypothesis test demonstrated that attitudes toward politicians had a stronger relationship to attitudes toward politicians who were men than politicians who were women (*r*_*diff*_ = 0.09, 99.5% CI [.05,.14]). See Fig 2. Notably, the analyses using the full sample produced similar statistics, *r*_*diff*_ = .07, 99.5% CI [.04,.10].

**Fig 3:**
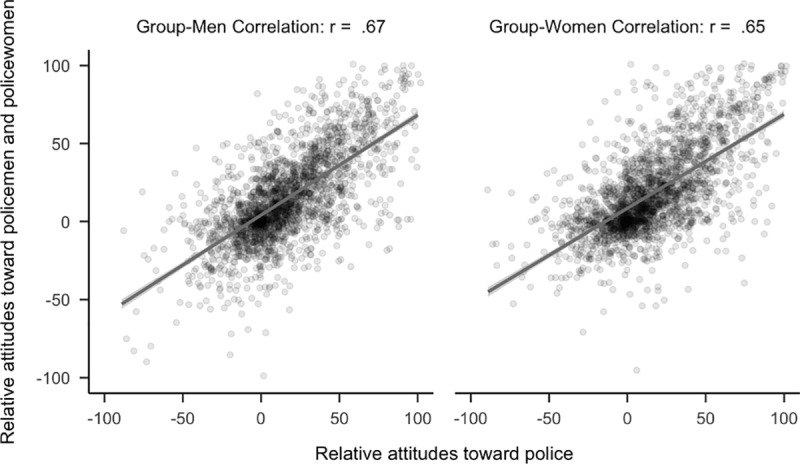
Plot of correlational differences for police officers. Side by side comparison of how attitudes toward police officers correlated with attitudes toward policewomen and policemen. The sample is the first 2,300 participants to complete the study.

**Fig 4:**
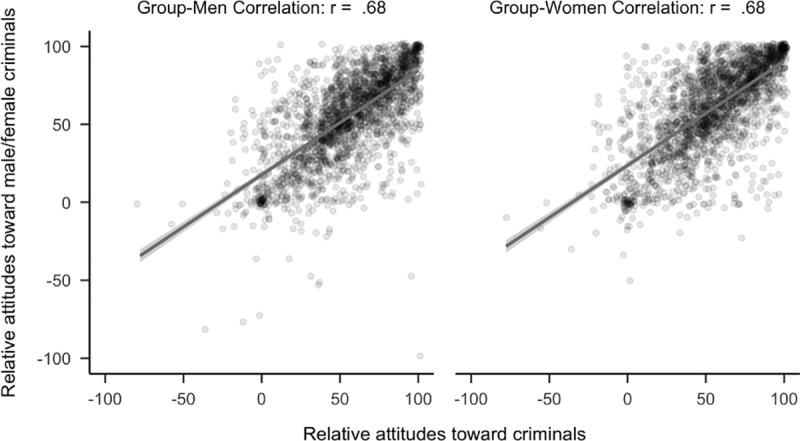
Plot of correlational differences for criminals. Side by side comparison of how attitudes toward criminals correlated with attitudes toward women and men who are criminals. The sample is the first 2,300 participants to complete the study.

Contrary to our predictions, confirmatory hypothesis tests found that attitudes toward police officers (*r*_*diff*_ = 0.03, 99.5% CI [−0.01, 0.06]) and criminals (*r*_*diff*_ = −0.01, 99.5% CI [−0.04, 0.03]) did not have a stronger relationship with attitudes toward the men rather than the women of the group (see Figs 3 and 4). Using the full sample, attitudes toward police officers (*r*_*diff*_ = −0.01, 99.5% CI [−0.03, 0.02] and criminals (*r*_*diff*_ = 0.02, 99.5% CI [−0.01, 0.04]) showed similar patterns.

### Regional endorsement of traditional gender roles as moderator

**Fig 5:**
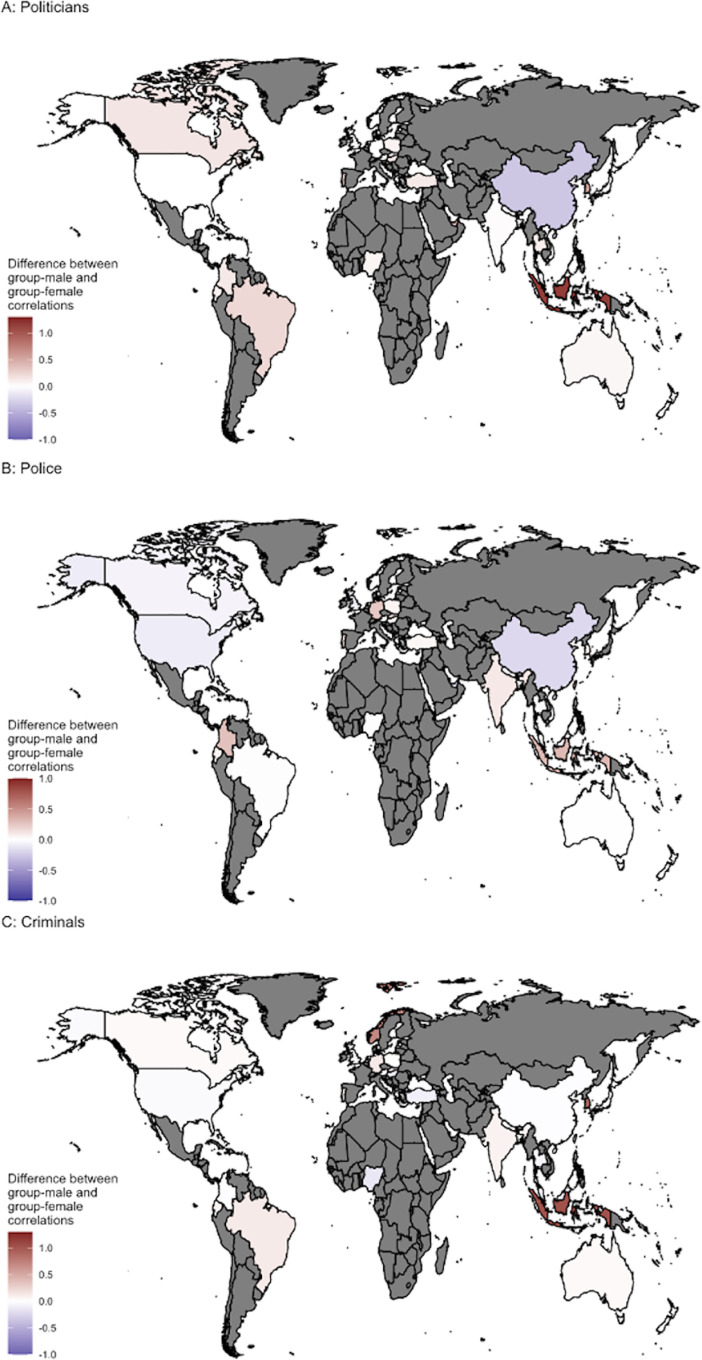
Plot of regional attitude differences. Regional differences in whether group attitudes are more strongly related to attitudes toward the men or women of the group for (A) attitudes toward politicians, (B) attitudes toward police, and (C) attitudes toward criminals. The maps used in this manuscript were created with the maps package in R, which uses the Natural Earth dataset in the public domain.

Fig 5 displays regional breakdowns for these effects toward traditionally ‘male’ groups. To test whether these effects were moderated by the extent to which a region endorses traditional gender roles, we conducted exploratory analyses on the correlation between the average difference in Group-Women attitudes and Group-Men attitudes in each country in our dataset and the extent to which that region agreed with traditional gender roles [35]. The extent to which a region endorsed traditional gender roles was positively related to gendered attitudes toward politicians (*r* = .43, 99.5% CI [.19,.80]), police officers (*r* = .31, 99.5% CI [.31,.75]), and criminals (*r* = .38 99.5% CI [.24,.78]). Individuals in regions that agreed with traditional gender roles more strongly showed larger differences in their attitudes for men versus women in traditionally male roles, although the magnitude of this relationship is uncertain.

### ‘Man’ as default in attitudes toward racial groups

**Fig 6:**
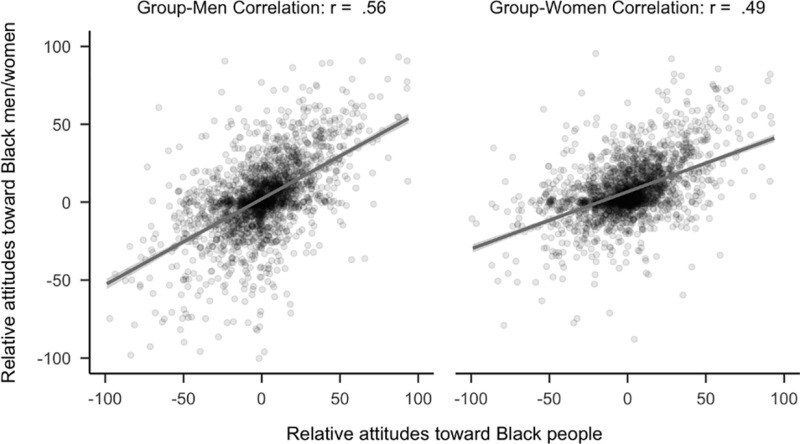
Plot of correlational differences for Black people. Side by side comparison of how attitudes toward Black people correlated with attitudes toward Black men and women. The sample is the first 2,300 people (median age = 20, age range 18 to 67) to complete the study that did not identify as Black.

For the following confirmatory hypothesis tests related to race, participants who were members of the target racial group were excluded from analyses. As predicted, attitudes toward Black people (*r*_*diff*_ = 0.07, 99.5% CI [0.02, 0.12]) had a stronger relationship to attitudes toward Black men rather than Black women. See Fig 6. The pattern is similar when using the full sample, *r*_*diff*_ = 0.08, 99.5% CI [0.05, 0.12].

**Fig 7:**
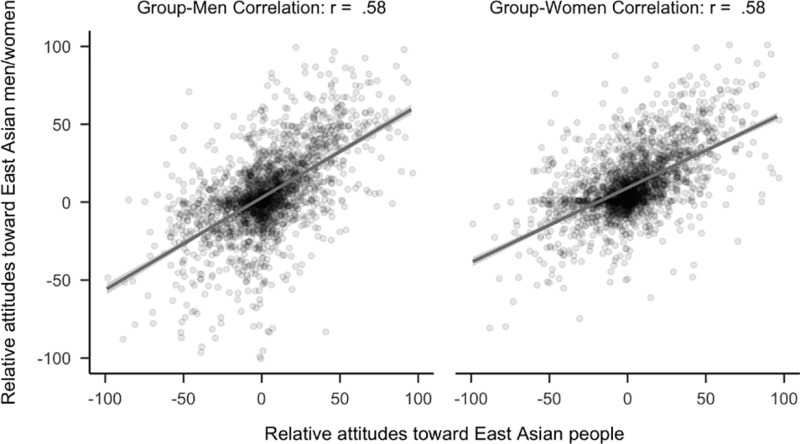
Plot of correlational differences for East Asian people. Side by side comparison of how attitudes toward East Asian people correlated with attitudes toward East Asian men and women. The sample is the first 2,300 people (median age = 20, age range 18 to 67) to complete the study that did not identify as East Asian.

However, attitudes toward East Asian people did not have stronger relationships to attitudes toward the men rather than the women of that group. See Fig 7. Specifically, attitudes toward East Asian people were not more strongly related to attitudes toward East Asian men or women, (*r*_*diff*_ = 0.01, 99.5% CI [−0.03, 0.05]). Notably, analyses using the full sample showed that attitudes toward East Asian people were more strongly related to attitudes toward East Asian men rather than East Asian women, *r*_*diff*_ = 0.03, 99.5% CI [0.00, 0.07].

**Fig 8:**
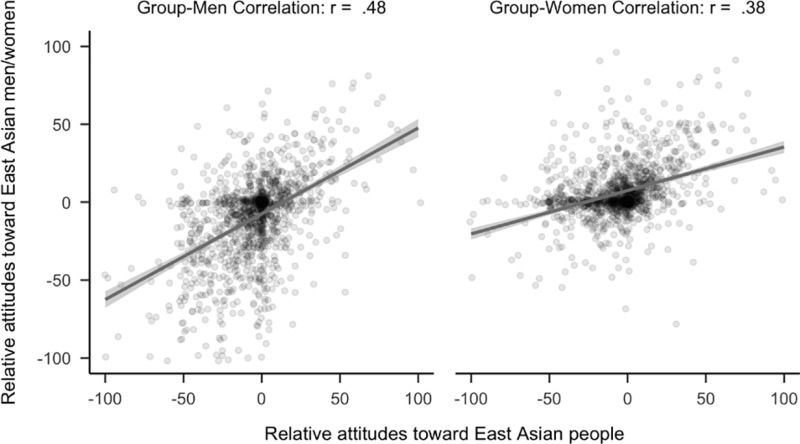
Plot of correlational differences for East Asian people for non-East Asian participants. Side by side comparison of how attitudes toward east Asian people correlated with attitudes toward East Asian men and women. The sample is the 1305 participants (median age = 19, age range 18 to 36) who completed the study in the U.S. and did not identify as East Asian.

We decomposed this analysis by participant location to examine our a priori hypothesis that individuals in the U.S. would view ‘woman’ as the default East Asian person. A confirmatory hypothesis test among U.S. participants who were not East Asian women demonstrated that attitudes toward East Asian people were more strongly related to attitudes toward East Asian men than attitudes toward East Asian women (*r*_*diff*_ = 0.10, 99.5% CI [0.02, 0.18]). However, because there were fewer than 2,300 participants in this analysis (*N* = 1305), this comparison was underpowered based on our initial threshold of *N* = 2,300 and is considered exploratory. See Fig 8.

**Fig 9:**
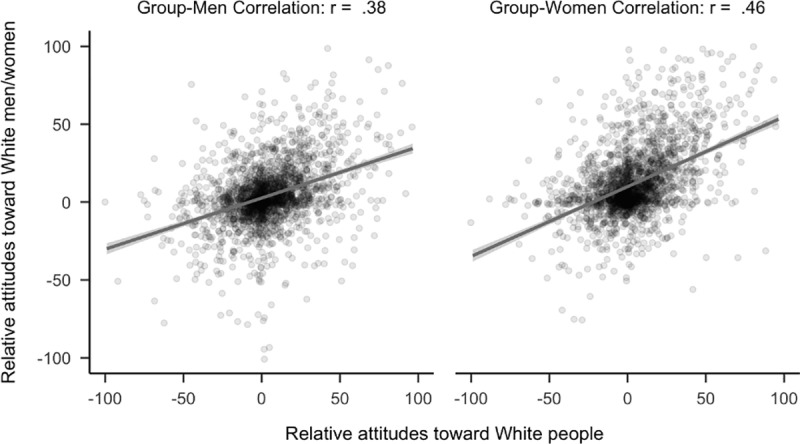
Plot of correlational differences for White people. Side by side comparison of how attitudes toward White people correlated with attitudes toward White men and women. The sample is the 2,260 participants (median age = 20, age range 18 to 67) who completed the study and did not identify as White.

Contrary to predictions, attitudes toward White people had a stronger relationship to attitudes toward White women than attitudes toward White men (*r*_*diff*_ = −0.08, 99.5% CI [−0.14, −0.02]). There were fewer than 2,300 non-White respondents (*N* = 2,260), thus, this comparison was underpowered based on our initial threshold of *N* = 2,300 and is considered exploratory. See Fig 9. Analyses were similar when participants who identified as “Turk” were categorized as “White”, *r*_*diff*_ = −0.10, 99.5% CI [−0.16, −0.04]. Notably, this latter analysis meets our initial threshold for confirmatory analyses.

**Fig 10:**
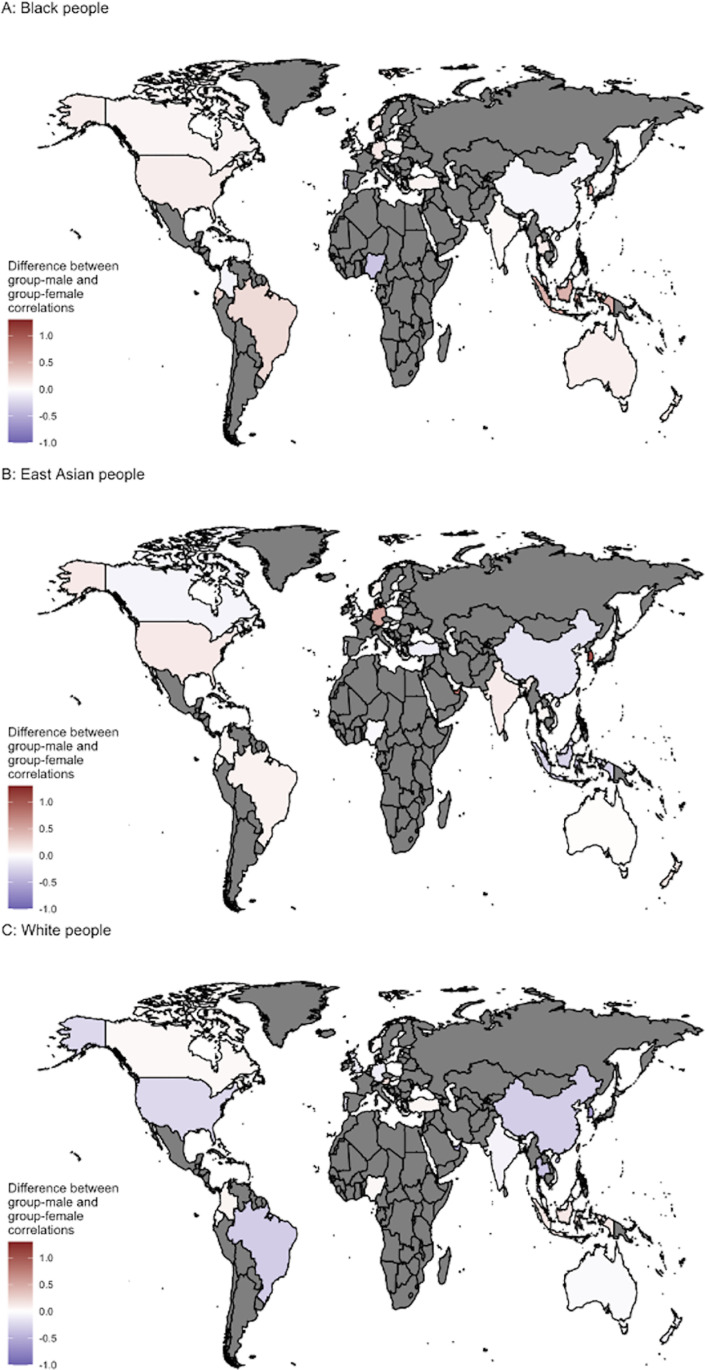
Plot of regional differences in attitudes. Regional differences in whether group attitudes are more strongly related to attitudes toward the men or women of the group for (A) attitudes toward Black people, (B) attitudes toward East Asian people, and (C) attitudes toward White people. The maps used in this manuscript were created with the maps package in R, which uses the Natural Earth dataset in the public domain.

Fig 10 presents regional breakdowns for the race data. We conducted exploratory analyses to examine whether ‘man’ as default in racial groups was related to the extent a region endorses traditional gender roles. The extent to which a region endorsed traditional gender roles had no clear relationship to more gendered attitudes toward Black people (*r* = .02, 99.5 CI [.55,.58]), White people (*r* = .09, 99.5 CI [.51,.62]), and East Asian people (*r* = −.11, 99.5 CI [.49,.63]). Unexpectedly, the relationship to East Asian people ran in the opposite direction such that the more a region endorsed traditional gender roles the more likely participants in that region had gendered attitudes toward East Asians, but confidence bounds are very wide, so the magnitude of these relationships is unclear.

### ‘Man’ as default in attitudes among women participants

**Fig 11:**
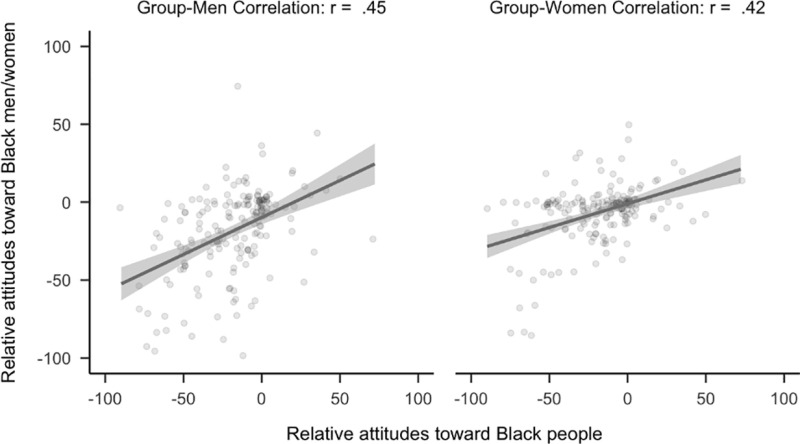
Plot of correlational differences for Black people in women. Side by side comparison of how attitudes toward Black people correlates with attitudes toward Black men and women. The sample is the 180 participants (median age = 20, age range 18 to 37) who completed the study and identified as Black women.

**Fig 12:**
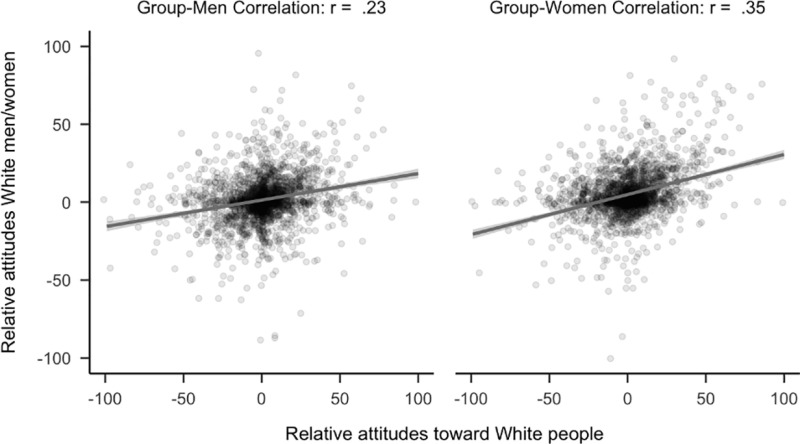
Plot of correlational differences for White people in women. Side by side comparison of how attitudes toward White people correlates with attitudes toward White men and women. The sample is the 2,152 participants (median age = 20, age range 18 to 64) who completed the study and identified as White women.

Exploratory analyses demonstrated that Black (*N* = 180) and White women (*N* = 2,152) did not view ‘man’ as the default member of their respective racial groups. Among Black women, there was no evidence that attitudes toward Black people had a stronger relationship to either attitudes toward Black men or Black women (*r*_*diff*_ = 0.03, 99.5% CI [−0.18, 0.24]). Similarly, among White women, attitudes toward White people were not more strongly related to attitudes toward White men. On the contrary, it was more related to attitudes toward White women (*r*_*diff*_ = −0.12, 99.5% CI [−0.19, −0.05]) as was found for non-White participants. See Figs 11 and 12. A similar pattern was found when participants who identified as “Turk” were categorized as “White”, *r*_*diff*_ = −0.10, 99.5% CI [−0.17, −0.03].

The latter effect is consistent with the unexpected finding reported above that among non-White participants, attitudes toward White people had a stronger correlation with attitudes toward White women than White men. Exploratory analyses revealed a non-significant difference in the same direction among the 690 participants that identified as White men, *r*_*diff*_ = −0.06, 99.5% CI [−0.18, 0.06].

**Fig 13:**
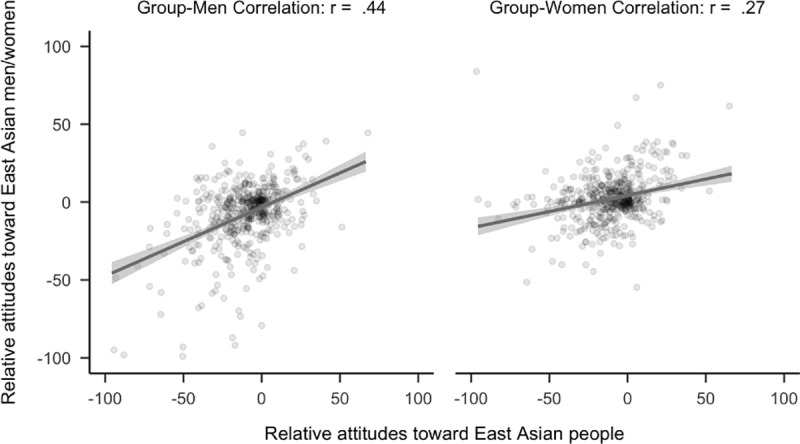
Plot of correlational differences for East Asian people in women. Side by side comparison of how attitudes toward East Asian people correlates with attitudes toward East Asian men and women. The sample is the 485 participants (median age = 20, age range 18 to 42) who completed the study and identified as East Asian women.

However, unexpectedly, exploratory analyses among East Asian women (*r*_*diff*_ = 0.16, 99.5% CI [0.02, 0.30]), attitudes against East Asian people were more strongly related to attitudes against East Asian men. See Fig 13.

## Discussion

The present research provides evidence for the extent and limitations of ‘man’ as default in attitudes toward multiple social groups among a diverse sample of participants recruited via a globally distributed laboratory network. Specifically, we found evidence suggesting ‘man’ as default in attitudes toward Black people and politicians. However, our findings did not support ‘man’ as default in attitudes toward police officers, criminals, White people or East Asian people which is inconsistent with previous work demonstrating the ubiquity of androcentrism [4]. However, the practical relevance of these effects is limited by the fact that all effect sizes were small.

However, the magnitude and direction of these effects varied by region (see Figs 5 and 10) and participant characteristics. For example, attitudes toward men politicians were the default on average when looking at the whole sample, they were not among participants in the U.S. or China. Chinese participants, in fact, showed a flipped direction of the effect. Similarly, though Black men were the default in the pooled sample, they were not among Black women or participants in Nigeria (See Fig 10). Thus, ‘man’ as default in attitudes likely depends on many contextual factors. We found some support for one contextual factor—the extent to which a region endorses traditional gender roles. Specifically, participants from regions that endorsed traditional gender roles were more likely to view men as default in attitudes, but the magnitude is uncertain due to endorsement of traditional gender roles being assessed by proxy. Future studies with individual-level assessment of endorsement of traditional gender roles are required to assess this potential effect more precisely.

Though there was regional variation in the results related to White people, the police, and criminals, it is surprising that attitudes toward the men of those groups were not default given past research demonstrating White men as default in the mental representation of White people [3] and the over-representation of men among the police [26] and criminals [28]. Future research should investigate the possibility that the present research’s reliance on positive dimensions of attitudes (warmth, positivity, likability, and favorability), increased the salience of attitudes toward women within those groups. For example, warmth and likability are communal traits [20,21] that may be more closely tied to attitudes toward the women of a group. Future research could probe this possibility by testing whether the default gender in attitudes is moderated by attitude valence.

Though the sample was diverse in terms of the number of regions (N > 40) represented, it comprised undergraduates and, therefore, may not represent the general population in any region. For example, undergraduate samples may be younger and more liberal than a region’s population. Additionally, the region with the most participants was the U.S. (N = 1422), so the opinions of participants from that region might be overrepresented in the overall sample. Future research should investigate the extent to which these factors (e.g., region, group membership, regional endorsement of traditional gender roles, and sampling) contribute to ‘man’ as default in attitudes.

## Conclusions

In conclusion, we used a methodology based on the measurement of attitudes to assess the extent to which ‘man’ is seen as default in three traditionally male social groups and three racial groups. Although ‘man’ was the default for attitudes toward Black people, that, unexpectedly, was not the case for attitudes toward White people. These findings speak to unique forms of prejudice that women experience either because they have ignored societal expectations for gender roles or because of their unique place in the intersection of race and gender.
